# No Evidence for Seed Transmission of Tomato Yellow Leaf Curl Sardinia Virus in Tomato

**DOI:** 10.3390/cells10071673

**Published:** 2021-07-02

**Authors:** Saeid Tabein, Laura Miozzi, Slavica Matić, Gian Paolo Accotto, Emanuela Noris

**Affiliations:** 1 Institute for Sustainable Plant Protection, National Research Council of Italy, Strada delle Cacce, 73, 10135 Torino, Italy; s.tabein@scu.ac.ir (S.T.); slavica.matic@ipsp.cnr.it (S.M.); gianpaolo.accotto@ipsp.cnr.it (G.P.A.); 2 Department of Plant Protection, Faculty of Agriculture, Shahid Chamran University of Ahvaz, Ahvaz 61349, Iran

**Keywords:** seed transmission, geminiviruses, reproductive tissue, tomato, embryos

## Abstract

Seed transmission is an important factor in the epidemiology of plant pathogens. Geminiviruses are serious pests spread in tropical and subtropical regions. They are transmitted by hemipteran insects, but a few cases of transmission through seeds were recently reported. Here, we investigated the tomato seed transmissibility of the begomovirus *tomato yellow leaf curl Sardinia virus* (TYLCSV), one of the agents inducing the tomato yellow leaf curl disease, heavily affecting tomato crops in the Mediterranean area. None of the 180 seedlings originating from TYLCSV-infected plants showed any phenotypic alteration typical of virus infection. Moreover, whole viral genomic molecules could not be detected in their cotyledons and true leaves, neither by membrane hybridization nor by rolling-circle amplification followed by PCR, indicating that TYLCSV is not a seed-transmissible pathogen for tomato. Examining the localization of TYLCSV DNA in progenitor plants, we detected the virus genome by PCR in all vegetative and reproductive tissues, but viral genomic and replicative forms were found only in leaves, flowers and fruit flesh, not in seeds and embryos. Closer investigations allowed us to discover for the first time that these embryos were superficially contaminated by TYLCSV DNA but whole genomic molecules were not detectable. Therefore, the inability of TYLCSV genomic molecules to colonize tomato embryos during infection justifies the lack of seed transmissibility observed in this host.

## 1. Introduction

The *Geminiviridae* family, with nine accepted genera and an increasing number of unassigned species, is the largest family of plant-infecting viruses [1]. Their small circular single-stranded genomic DNA (ssDNA) is encapsidated into twinned icosahedral particles and replicates through double-stranded DNA (dsDNA) intermediates in the nucleus [2]. Within geminiviruses, the *Begomovirus* genus is the largest genus of plant viruses and includes more than 400 assigned species [1]; it counts etiological agents of several diseases that affect economically important crops, such as cotton, cassava, tomato, potato and pepper [3]. 

Until now, geminiviruses have been commonly believed to be naturally transmitted by hemipteran insects, such as whiteflies, leafhoppers, plant-hoppers and aphids. Recently, seed transmissibility of some begomoviruses, such as *sweet potato leaf curl virus* [4], *tomato yellow leaf curl virus* (TYLCV) [5], *tomato leaf curl New Delhi virus* [6,7], *mung bean yellow mosaic virus* [8], *bitter gourd yellow mosaic virus* [9] and *pepper yellow leaf curl Indonesia virus* [10] has been reported. This feature has been evoked to clarify the spread of certain viruses in specific areas, especially where insect vectors are not reported [5,11] or to elucidate geminivirus origin, evolution and distribution patterns [11]. Furthermore, the seed transmissibility of TYLCV, one of the agents of the tomato yellow leaf curl disease (TYLCD) in tomato crops, has been pointed out as an additive factor that hampers the management of the disease, coupled with the increased presence of its vector, the whitefly *Bemisia tabaci* [5]. However, the seed transmissibility of TYLCV and other begomoviruses has been recently questioned [12,13,14,15] and remains controversial. 

*Tomato yellow leaf curl Sardinia virus* (TYLCSV), another monopartite begomovirus, is present in the entire Mediterranean basin [16] and, in association with TYLCV and other TYLCV-like viruses, contributes to the worldwide spread of TYLCD [17]. Clarifying if other viruses inducing TYLCD are seed-transmissible is particularly useful for the seed industry, to define at which level phytosanitary measures must be adopted to prevent uncontrolled spread of the diseases they induce. In the present study, we explored the seed transmissibility of TYLCSV in the susceptible tomato cultivar Moneymaker, evaluating the presence of its genomic and replicative forms in the progeny of infected plants. Moreover, we assessed if this virus reaches the vegetative and, particularly, the reproductive organs of tomato plants during infection, focusing on the identification of whole viral molecules in the embryos. 

## 2. Materials and Methods

### 2.1. Preparation of Biological Material

**Figure 1:**
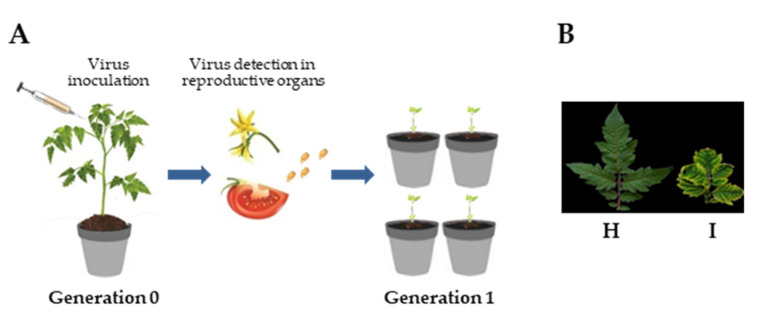
Analysis of the seed transmissibility of TYLCSV in tomato plants. (**A**) Scheme of the experiment to investigate the presence of TYLCSV in tissues of reproductive organs of agroinfected plants (Generation 0) and its transmissibility to progeny plants (Generation 1). (**B**) Tomato yellow leaf curl disease symptoms on tomato leaves at 6 weeks post-inoculation (H, healthy; I, infected).

Tomato plants (cv. Moneymaker) were inoculated at the four-leaf stage by *Agrobacterium tumefaciens* LBA4404 cultures harboring a 1.8mer TYLCSV construct (Genbank Acc. No. X61153) [18], while another set of plants received bacterial cultures containing an empty vector, as control. Plants were maintained in an insect-proof greenhouse at 20–28/16–20 °C (day/night) (Generation 0, G_0_; Figure 1A). After symptom development, samples of leaves and different reproductive tissues, i.e., seeds, embryos, petals, sepals, pistils and, stamens, from these plants were taken for further analyses. Mature fruits were collected from the same plants, and seeds were separated from the fruit flesh. After several washes with sterile water, the seeds were dried at room temperature and stored at 4 °C. Seeds derived from TYLCSV-inoculated plants or from mock-inoculated plants were used for two independent grow-out tests (Generation 1, G_1_). Seeds collected from eight infected plants were surface-sterilized for 10 min in 70% ethanol, then left for 10 min in 10-fold diluted commercial bleach, followed by several washes in sterile water before sowing. For analysis on G_1_ seedlings, 20 to 24 seeds from each G_0_ plant were used. G_1_ plants were maintained in an insect-free greenhouse and monitored for the appearance of symptoms during their life cycle (Figure 1A). Cotyledon and leaf samples collected at the 5–6 leaf stage were subjected to total DNA extraction (see below) and tested for the presence of TYLCSV DNA. 

### 2.2. Nucleic Acid Extraction

Total DNA was extracted by the dot-blot method [19] from all samples, using, in all cases, 100 mg of tissue. In the case of whole seed and embryo tissue analysis, seeds were previously sterilized with 70% ethanol for 10 min, followed by 20 min in 10-fold diluted commercial bleach and three washes with distilled water, to exclude superficial contaminations. In the case of embryos, 5 to 10 seeds were incubated in the dark at room temperature in 9 cm Petri dishes, over two layers of moistened filter paper. After swelling, embryos were separated from seed coats using a razor blade, and total DNA was extracted. In some experiments, freshly dissected embryos were further treated for 5 min with 10-fold diluted commercial bleach, followed by extensive washing with sterile distilled water. 

### 2.3. Virus Detection and Quantification

#### 2.3.1. End-Point PCR

PCR assays were carried out in 25 µL reactions containing 1× PCR buffer, 200 µM each of dNTPs, 0.4 µM of primers (Table 1), 2 mM MgCl_2_ and 1 unit per µL Platinum Taq DNA polymerase (Invitrogen, Thermo Fisher Scientific, Waltham, MA, USA). The mixture was denatured for 4 min at 95 °C, followed by 35 cycles of 30 s at 95 °C, 30 s at 58 °C and 30 s at 72 °C and by a final cycle at 72 °C for 10 min. The mixture was loaded onto 1% agarose gels, and the gels were run at 100 V, stained with ethidium bromide and visualized under UV light.

#### 2.3.2. Quantitative Real-Time PCR

Quantitative real-time PCR (qPCR) was carried out using iCycler iQTM Real-Time PCR Detection System (BioRad Laboratories, Hercules, CA, USA), as described previously [22], with the following cycling parameters: 1 cycle at 50 °C for 3 min; 1 cycle at 95 °C for 5 min; 45 cycles, each consisting of 15 s at 95 °C and 1 min at 60 °C. A melting curve was recorded at the end of each run to assess amplification specificity. All reactions were performed with three technical replicates. PCR efficiency was calculated using standard curves constructed with serial dilutions of DNA extracted from infected plants. Data acquisition and analysis were handled by the BioRad iCycler software (version 3.06070) that calculates Ct values and standard curves. The primer pair TY2222(+)/TY2371(−) (Table 1) was used to amplify TYLCSV genomic fragments, while the primer pair SlyAPX-862(+)/SlyAPX-948(−) (Table 1) was used for the amplification of the tomato gene Y16773.1 coding for ascorbate peroxidase (APX), utilized as reference gene. The relative virus amount was estimated according to [20]. 

#### 2.3.3. Southern Blot Assay

DNA samples (approximately 300–500 ng) were loaded onto 1% agarose gel and separated in 0.5 × TBE, containing 0.5 µg/mL ethidium bromide, electrophoresed at 70 V for 3 h and blotted onto positively charged nylon membranes (Roche, Basel, Switzerland). Membranes were then hybridized with a digoxygenin-labeled coat-protein-specific probe obtained with the TY1(+)/TY2(−) primers (Table 1), following the manufacturer’s instructions (Roche, Basel, Switzerland). 

#### 2.3.4. Rolling Circle Amplification (RCA)

DNA extracted from G_1_ material (cotyledons and true leaves) was diluted 1 to 5 in water and subjected to RCA using the TempliPhi Kit (GE Healthcare Life Science, Boston, MA, USA), according to the manufacturer’s instructions. A DNA extract from an infected tomato plant was used as positive control, while extracts derived from G_1_ seedlings obtained from a healthy plant were used as negative controls. RCA products (diluted 1 to 5 in water) were used for end-point PCR amplification.

## 3. Results

### 3.1. TYLCSV DNA Is not Seed-Transmitted to the Progeny of Infected Plants

To investigate the seed transmissibility of TYLCSV, tomato plants were agroinoculated with a TYLCSV clone and maintained under insect-free conditions until flower production and fruit maturation (Figure 1A). Mild curling and yellowing of the leaflet edge were manifest on inoculated plants at three weeks post inoculation (wpi) and by six wpi, typical symptoms, including severe leaf curling, cupping and yellowing were evident on all newly emerged leaves (Figure 1B). 

**Figure 2:**
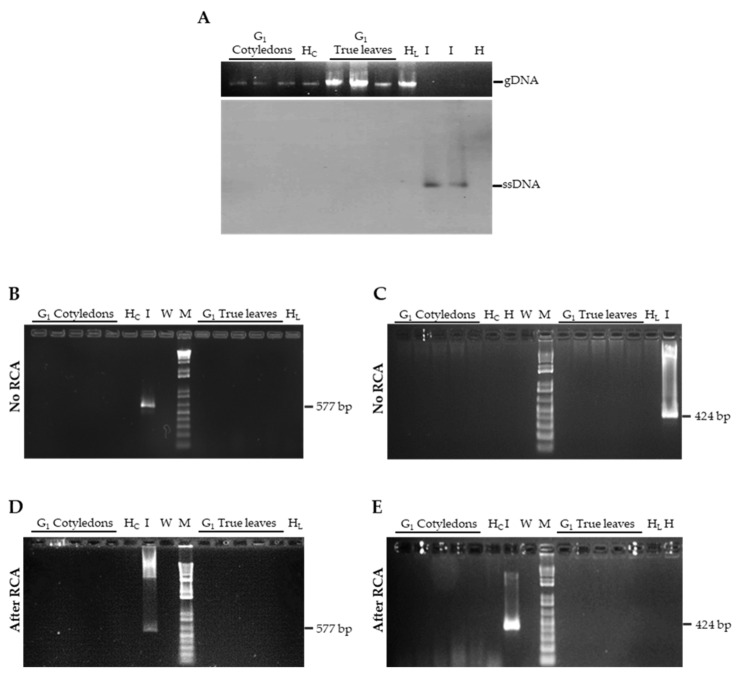
Detection of TYLCSV DNA in G_1_ seedlings. (**A**) Southern blot analysis on cotyledon and true leaf extracts from TYLCSV-infected plants. H_C_ and H_L_ extracts from cotyledon or true leaves of the G_1_ seedlings of healthy plants, respectively. I, DNA extract (3–5 ng) from an infected plant, used as positive control. gDNA, host genomic DNA, shown as loading control. ssDNA, circular single stranded genomic DNA; (**B**,**C**) PCR analysis of cotyledon and true leaf extracts from TYLCSV-infected plants, performed using the TY1(+)/TY2(−) (**B**) or the TY2458(+)/TY109(−) (**C**) primer pairs. (**D**,**E**) PCR analysis of RCA products obtained from the same cotyledon and true leaf extracts of B and C, performed with the TY1(+)/TY2(−) (**D**) or the TY2458(+)/TY109(−) (**E**) primer pairs. H_C_ and H_L_, negative controls as above; H, healthy plant, negative control; I, infected plant, positive control; W, water control; M, 1 kb Plus DNA Ladder (Invitrogen, Thermo Fisher Scientific, Waltham, MA, USA).

Seeds collected from eight infected G_0_ plants, deriving from two independent inoculation experiments, were used in two separate grow-out assays (Figure 1A). The derived G_1_ seedlings (*n* = 180) were analyzed for their phenotype and for the presence of viral DNA. Overall, none of the plants displayed any symptom that could be ascribed to TYLCSV infection, at least up to two months after sowing. When the presence of viral DNA was investigated in cotyledon and true leaf extracts of G_1_ seedlings, using a Southern blot developed with a virus-specific probe targeting the coat protein gene, viral genomic forms could not be detected (Figure 2A), even when samples were concentrated up to 20-fold and the membrane was exposed for prolonged time (not shown). Since membrane-based detection of TYLCSV is about 10^3^ times less sensitive than PCR [19], we decided to carry out a series of PCR experiments using primers targeting two different regions of the viral genome, i.e., TY1(+)/TY2(−) specific for the coat protein gene and TY2458(+)/TY109(−) amplifying a portion of the C1 gene and of the intergenic region. However, in none of the cotyledon or true leaf samples from both experiments were TYLCSV-related amplicons obtained (Figure 2B,C and Supplementary Figure S1). 

To increase the sensitivity and particularly, the selectivity of our assays, we subjected the samples to RCA, a reaction that specifically targets circular genomic molecules; RCA products were then used as templates for PCR with the virus-specific primers TY1(+)/TY2(−). However, even in this case, no TYLCSV-related amplicons could be visualized (Figure 2D,E and Supplementary Figure S2). 

Taken together, these results indicate that TYLCSV DNA cannot be detected in G_1_ seedlings or, at least, that its amount is below the detection limits of the adopted techniques, suggesting that it is not seed-transmissible in tomato. 

### 3.2. Genomic TYLCSV DNA Reaches Reproductive Organs of Tomato Plants, but Is Unable to Invade Embryos

**Figure 3:**
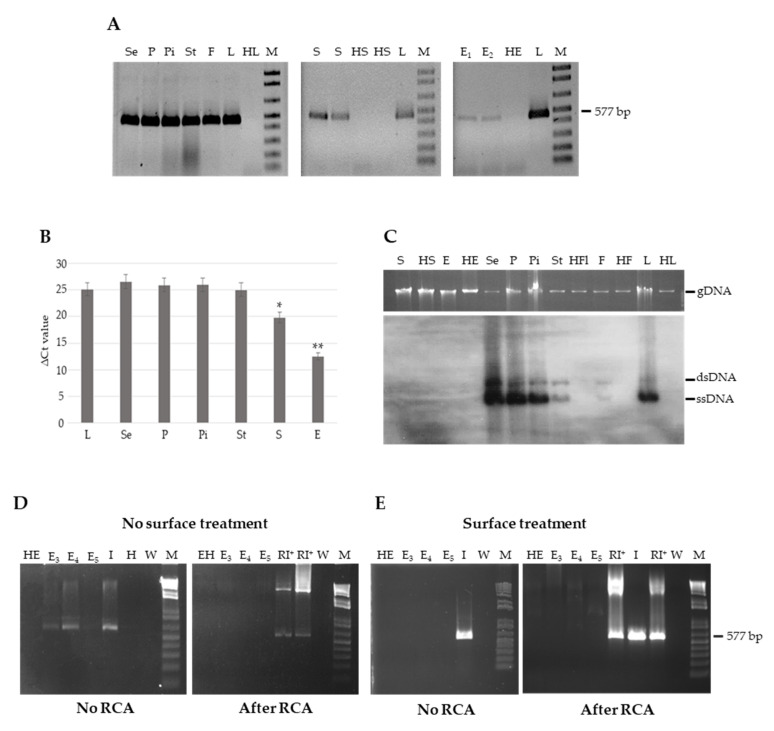
Detection of TYLCSV DNA in different tissues of infected tomato plants. (**A**) PCR analysis using the TY1(+)/TY2(−) primers. (**B**) Quantification of TYLCSV DNA by qPCR. The Y-axis indicates the ΔCt value, calculated as follows: ΔCt = |Ct_tissueTYLCSV_− Ct_SlyAPX_|. Asterisks indicate significant differences (Kruskal–Wallis test, *p*-value < 0.05). (**C**) Southern blot analysis with a coat-protein-specific probe. ssDNA and dsDNA, TYLCSV single-stranded genomic and double-stranded replicative forms, respectively; gDNA, host genomic DNA loading, shown as control. (**D**,**E**) PCR analysis of embryos (*n* = 18–20, from three different plants) extracted immediately after dissection (No surface treatment) or following a sterilization step (Surface treatment). PCRs were performed directly on DNA samples (No RCA) or on RCA products obtained with the same extracts (After RCA). Unless indicated, all samples were collected from infected plants and from surface sterilized seeds. Se, sepals; P, petals; Pi, pistils; St, stamens; F, fruits; L, leaves; HL, healthy leaves; S, seeds; HS, healthy seeds; E_1–5_, embryos (five different batches); HE, healthy embryos; HFl, healthy flowers; HF, healthy fruits; I, infected plant, positive control; H, healthy plant, negative control; RI^+^, RCA on infected plant, positive control (two different samples); W, water control; M, 1 kb Plus DNA Ladder (Invitrogen, Thermo Fisher Scientific, Waltham, MA, USA).

The unsuccessful detection of viral DNA in the progeny of infected plants prompted us to investigate if TYLCSV was able to invade the tissues associated with reproductive organs during infection, a mandatory ability for the transmission of the virus to the progeny. For this, we extracted DNA from different organs of G_0_ infected plants, such as petals, sepals, pistils, stamens, fruit flesh, seeds and embryos, and subjected them to end-point PCR analysis using the TY1(+)/TY2(−) primers. Positive signals were obtained from leaves and extracts prepared from all the organs considered (Figure 3A). To quantify the viral DNA present in each sample, a qPCR analysis was conducted, showing overall no statistically significant differences among the different organs, except for whole seeds and embryos that harbored approximately 10 and 10^3^ times less viral DNA compared to leaf tissue, respectively (Kruskal–Wallis test, *p*-value < 0.05) (Figure 3B and Supplementary Table S1). These indicate that viral DNA can be amplified from all the organs, and that a decreasing gradient of concentration exists from the vegetative to the reproductive organs. However, this cannot prove the integrity of the genomic viral DNA, a prerequisite for seed transmissibility. To this aim, we first carried out a Southern blot hybridization analysis, showing that genomic and replicative forms (ssDNA and dsDNA, respectively) are present in all extracts, except seeds and embryos, for which no signals could be recorded (Figure 3C). 

The reduced concentration of TYLCSV DNA in seeds and embryos detected by qPCR and the inability to detect genomic or replicative forms by a membrane-based approach indicated a poor accumulation, if any, of viral genome in these organs. Therefore, we adopted the RCA-based approach above used to identify circular genomic DNAs in G_1_ seedlings. For this, three new batches of embryos (*n* = 18–20 each) were collected from infected seeds. Once verified by PCR that TYLCSV DNA could be amplified from all extracts (Figure 3D, left panel), the RCA/PCR procedure was carried out. Amplification occurred only in the positive control consisting of an infected leaf sample, while no bands resulted from the embryos (Figure 3D, right panel), confirming the results obtained by Southern blot and in agreement with the lack of seed transmissibility observed. 

This prompted us to verify if the amplification products obtained from embryos by direct PCR, without performing RCA, could result from a contamination of the external layers of the embryos by the surrounding tissues. For this, embryos were subjected to a surface-sterilization step and their DNA extracts were processed by PCR and RCA/PCR, as above. Notably, following such surface treatment, it was possible to successfully amplify the endogenous 25S rRNA gene (Supplementary Figure S3), while the amplification of TYLCSV DNA was totally prevented by direct PCR, and no RCA/PCR products were obtained (Figure 3E, left and right panels, respectively). 

Overall, these results allow us to conclude that embryos dissected from infected seeds are surface-contaminated by TYLCSV DNA, originating from the surrounding maternal tissue during seed formation or during the processes of embryo dissection and manipulation. The most important result of these analyses is that no circular genomes of TYLCSV can be detected in embryos, neither in the external layers, nor in the inner tissues of such seed portions, implying that whole viral molecules do not reach embryos, in line with the observed lack of TYLCSV transmission through seeds. 

## 4. Discussion

Considering the importance of TYLCD and the continuous intercontinental spread of its vector to new areas [23], biological features related to its diffusion and etiology, including the seed-transmission of the viruses responsible for it, must be correctly evaluated. Indeed, recent reports describing the seed-transmissibility of a few begomoviruses changed previous attitudes about the propagation of these pathogens in agricultural and natural contexts, providing tentative justification for their intercontinental spread. This new concept prompted us to evaluate for the first time the potential seed transmissibility of TYLCSV in tomato, one of the most important vegetable crops worldwide, heavily affected by TYLCD. Actually, although TYLCD is caused by up to thirteen independent TYLCV-like viral species [24], only the seed transmissibility of TYLCV has been investigated so far; moreover, the results obtained with this viral species are controversial [5,12,13,14], highlighting the need to deepen our knowledge on this biological feature and to extend the investigation to other viruses responsible for such a disease complex. Among the TYLCD-inducing viruses, TYLCSV shares about 76–79% identity at the nucleotide level with TYLCV and differ from it, not only in terms of host range [25,26,27], but also in its response to the *Ty* genes-based resistance [28,29] and in the functionality of its encoded proteins in the silencing process or in the induction of pathogenic effects [30,31]. 

Our grow-out assays showed not only that the progeny of infected plants did not display viral symptoms, but also that TYLCSV DNA and, more importantly, whole genomic viral molecules are undetectable in the cotyledons and true leaves of G_1_ seedlings. These results are clearly in contrast with the 70 to 85% rates of seed transmissibility reported for TYLCV in tomato [5] and are rather in line with the conclusions reached by other groups for the same virus in tomato [13,14] and in *Nicotiana benthamiana*, a laboratory solanaceous host highly susceptible to TYLCV and supporting high viral accumulation [12]. It is worth noting that, considering the 70% infection rate of G_1_ seedlings reported for TYLCV DNA in tomato [5], the probability that we missed the detection of TYLCSV DNA in the progeny tested in our experiments is about 10^−94^, based on the binomial distribution calculation. This probability increases to 10^−18^ and 10^−8^ if we consider the lower infection rates of G_1_ seedlings reported for the same virus in other hosts, such as soybean and pepper, with infection rates of 21% [6] and 10% [32], respectively. Similar levels of probability are obtained if one considers the infection rates of other geminiviruses in different progeny plants [7,8,9,10,11]. Therefore, our data allow us to exclude seed transmission of TYLCSV, at least at the rates that were measured for TYLCV or for other begomoviruses.

It is worth mentioning that the experiments here reported were conducted with plants artificially inoculated with an infectious clone of TYLCSV [18], through a classical agrobacterium-mediated procedure. Although we cannot exclude that such method could mount plant responses different from those occurring during natural infections, it is relevant to note that previous studies investigating the seed-transmissibility of other begomoviruses explicitly reported that agroinoculation and natural infection have identical outcomes in terms of the localization of the viral genome and seed transmissibility [5,14].

Seed transmission depends on the ability of a virus to reach the reproductive organs of the host, facing the problem of crossing physical barriers of the different organs, invading different kinds of tissues and overcoming the physiological alterations that occur during seed maturation and storage. To elucidate such biological process and gain proof of the efficient localization of the pathogen in reproductive structures, it is important, not only to amplify portions of viral genomic sequences, but also to focus on the presence of whole viral genomes using suitable molecular techniques. In this work, we initially verified that TYLCSV does reach the reproductive organs of infected tomato plants, including flowers, fruits and seeds, and that, similarly to TYLCV [14], its titer significantly decreases in surface-sterilized seeds and, particularly, in embryos. However, in such organs, no circular viral genomes were detected, and, when embryos were surface sterilized, no viral DNA could be amplified any more, implying that these seed structures do not harbor intact viral genomes in an amount sufficient for detection through RCA/PCR and that they are surface-contaminated by viral DNA, which seems unpreserved. These results are in line with the strict-phloem-limited distribution of TYLCSV [32,33] and with the absence of known synaptic connection between mother plant cells and embryo tissue in tomato. It would be interesting to investigate if such connections exist in the case of the geminiviruses for which seed transmission has been reported, concentrating on the factors that allow a geminivirus to invade non-phloematic tissue.

Previous reports of geminivirus seed transmissibility often relied on the successful PCR amplification rates of the viral genome, even in the absence of symptoms [5,6,7,8,34], without assessing if whole viral genomic molecules or replicative forms are present, thus precluding fair comparison with our results. The same is true for virus-localization studies in seeds and embryos, often considered the proof for seed transmissibility. Indeed, TYLCV DNA has been detected in the embryos of infected tomato seeds using in situ hybridization [35], a technique that cannot selectively discriminate if whole molecules are present in the observed tissues.

Given the controversial results reported for TYLCV in tomato [5,12,13,14] and the lack of seed transmissibility here described for TYLCSV, it remains necessary to clarify if specific experimental or environmental conditions are involved, to identify the viral factors governing this process and to evaluate the seed-transmission behavior of the other TYLCV-like viruses responsible for the TYLCD [24]. Considering that abiotic/biotic stresses, including mixed infections, can contribute to promote viral accumulation and replication, it will be of paramount importance for the global tomato seed trade to further investigate the possible impact of these factors on seed transmission. Furthermore, it is crucial to verify if TYLCD agents do persist in the seeds of other tomato cultivars or in weeds growing around cultivated fields, further complicating the eradication of the disease, particularly in field conditions.

## Figures and Tables

**Table 1:** List of primers used in this study.

Primers	Sequence (5′ to 3′)	Size of Amplicon (bp)	Reference
TY1(+)	GCCCATGTA(T/C)CG(A/G)AAGCC	577	[20]
TY2(−)	GG(A/G)TTAGA(A/G)GCATG(A/C)GTAC		
TY2458(+)	CATTTTCATGTAGTTCTCTG	424	This manuscript
TY109(−)	CACCAGCTGAACAGTTATTTAA		
TY2222(+)	GTCGTTGGCTGTCTGTTGTC	150	[21]
TY2371(−)	AGGTCAGCACATTTCCATCC		
SlyAPX-862(+)	CCCCTTTTGGCTTAATACTCG	87	[21]
SlyAPX-948(−)	GCAGAAATGGAAATGCGATAA		

## Data Availability

All data are contained within the article or as Supplementary Materials.

## Supplementary Materials

The following are available online at https://www.mdpi.com/article/10.3390/cells10071673/s1, Figure S1: PCR analysis of Generation I; Figure S2: Detection of TYLCSV DNA in Generation 1 (G_1_) seedlings of the first grow out experiment; Figure S3: Detection of the endogenous 25S ribosomal RNA (rRNA) gene (Acc. No. NR_137326.1) in three different embryos batches (*n* = 18–20, from three different plants) dissected from seeds of TYLCSV-infected plants; Table S1: Raw data relative to the quantification of TYLCSV DNA by qPCR in tissues derived from different plant organs.
