# Supplementary material for: No Evidence for Seed Transmission of Tomato Yellow Leaf Curl Sardinia Virus in Tomato
Source: Cells. 2021 Jul 2;10(7):1673. doi: 10.3390/cells10071673 (PMC8306144; doi:10.3390/cells10071673)
Supplement: Supplementary file 1 [file cells-10-01673-s001.zip › cells-1252715-supplementary.pdf]

Tabein et al
Supplementary Table 1

Supplementary Table 1. Raw data relative to the quantification of TYLCSV DNA by qPCR in tissues derived from different plant organs (see Figure 3B).

Sample	Ct *TYLCSV*	Ct *apx*	ΔCt (*apx-TYLCSV*)	ΔCt Average
LEAF	8,18	33,13	24,95	25,187
LEAF	7,98	33,33	25,35	
LEAF	8,2	33,46	25,26	
PETAL	6,99	32,63	25,64	25,933
PETAL	6,4	32,64	26,24	
PETAL	7,01	32,93	25,92	
SEPAL	6,71	33,04	26,33	26,623
SEPAL	6,23	33,04	26,81	
SEPAL	6,31	33,04	26,73	
PISTIL	7,92	33,53	25,61	25,457
PISTIL	8,08	33,28	25,2	
PISTIL	7,84	33,4	25,56	
STAMEN	11,13	36,08	24,95	25,020
STAMEN	10,85	36,29	25,44	
STAMEN	11,51	36,18	24,67	
EMBRYO	23,82	36,18	12,36	12,467
EMBRYO	23,84	36,27	12,43	
EMBRYO	23,92	36,53	12,61	
SEED	15,12	34,82	19,7	19,773
SEED	15,2	35,15	19,95	
SEED	15,49	35,29	19,8	
SEED	14,3	34,18	19,88	
SEED	14,04	33,91	19,87	
SEED	14,59	34,03	19,44	

Tabein et al
Supplementary Figure 1

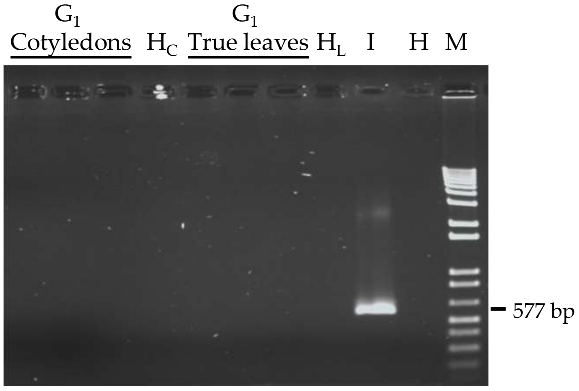

Supplementary Figure 1. PCR analysis of Generation 1 (G_1) seedlings of the first grow out experiment. PCR was conducted on bulked extracts from both cotyledon and true leaves from three different TYLCSV-infected plants, using primers TY1(+)/TY2(-). H_C and H_L represent extracts from cotyledon or true leaf samples derived from healthy plants. H and I, healthy and TYLCSV infected plant, used as negative and positive controls, respectively. M, 1kb Plus DNA Ladder (Invitrogen).

Tabein et al
Supplementary Figure 2

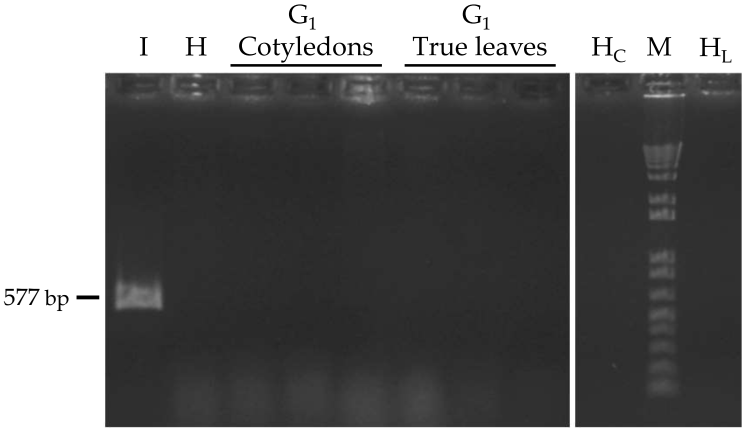

Supplementary Figure 2. Detection of TYLCSV DNA in Generation 1 (G_1) seedlings of the first grow out experiment. PCR was conducted with primers TY1(+)/TY2(-) on RCA products obtained from bulked DNAs extracted from cotyledon and true leaves of three different TYLCSV-infected plants (20 individual samples each). H_C and H_L, PCR on RCA products obtained from bulked cotyledon or true leaf samples of G1 seedlings of an healthy plant. I and H, DNA extracts from an infected/healthy plant used as positive/negative control, respectively. M, 1kb Plus DNA Ladder (Invitrogen).

Tabein et al
Supplementary Figure 3

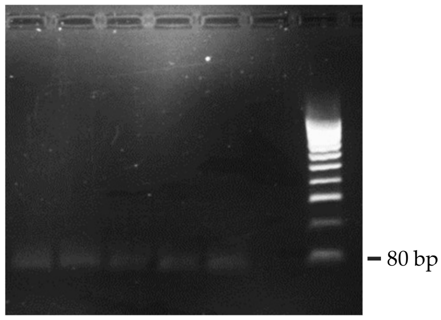

Supplementary Figure 3. Detection of the endogenous 25S ribosomal RNA (rRNA) gene (Acc. No. NR_137326.1) in three different embryos batches (n=18-20, from three different plants) dissected from seeds of TYLCSV-infected plants. Before DNA extraction, embryos were surface sterilized for 5 minute in 1:10 commercial bleach and extensively washed with sterile water. PCR was conducted with primers UNIV25S_rRNA(+) (5′-TCAGGTCTCCAAGGTGAACA-3′) and UNIV25S_rRNA(-) (5′-CCGAAGTTACGGATCCATTT-3′), generating an amplicon of 80 bp. HE, healthy embryos,; E_{3-5}, embryos (three different batches); I, infected plant, positive control; W, water control; M, HyperLadder™ 100bp (Bioline).
